# A clustered randomized control trial to assess feasibility, acceptability, and impact of implementing the birth companion intervention package in Ethiopia, Kenya, and Nigeria: study protocol

**DOI:** 10.1186/s12913-023-10082-w

**Published:** 2023-10-14

**Authors:** Della Berhanu, Gadise Bekele, Hanna Melesse, Felagot Taddese, Patricia Owira, Griffins Manguro, Oluwatosin Laleye, Zubaida Farouk, Mobolanle Balogun, Anne Hyre, Samuel Mwaura, Osborn Koech Kiptoo, Valentino Muyundo Wabwile, Siraj Mohammed, Konjit Wolde, Dedefo Teno, Eberechukwu Chinedu Eke, Jennyfer Oluyemisi Don-Aki, Lisa Noguchi, Stephanie Suhowatsky, Elizabeth Doggett, Gayane Yenokyan, Alemayehu Worku

**Affiliations:** 1 Jhpiego, Addis Ababa, Ethiopia; 2 Addis Continental Institute of Public Health, Addis Ababa, Ethiopia; 3 St. Paul’s Hospital Millennium Medical College, Addis Ababa, Ethiopia; 4 International Center for Reproductive Health Kenya, Mombasa, Kenya; 5 Ghent University, Faculty of Medicine and Health Sciences, Ghent, Belgium; 6 Maternal and Reproductive Health Research Collective, Lagos, Nigeria; 7 Centre for Infectious Diseases Research, Bayero University Kano, Kano, Nigeria; 8 Department of Community Health & Primary Care, College of Medicine, University of Lagos, Lagos, Nigeria; 9 Jhpiego, Baltimore, USA; 10 Jhpiego, Nairobi, Kenya; 11 Jhpiego, Abuja, Nigeria; 12 Johns Hopkins Bloomberg School of Public Health, Baltimore, USA

**Keywords:** Birth companion, Labor companion, Labor support, Experience of care, Maternal and neonatal mortality, Facility-based birth, Intrapartum care, Respectful maternity care, Quality of care

## Abstract

**Background:**

A birth companion is a simple and low-cost intervention that can improve both maternal and newborn health outcomes. The evidence that birth companionship improves labor outcomes and experiences of care has been available for many years. Global and national policies exist in support of birth companions. Many countries including Ethiopia, Kenya, and Nigeria have not yet incorporated birth companions into routine practice in health facilities. This paper presents the protocol for a trial that aims to assess if a package of interventions that addresses known barriers can increase the coverage of birth companions.

**Methods:**

This two parallel arm cluster randomized controlled trial will evaluate the impact of a targeted intervention package on scale-up of birth companionship at public sector health facilities in Ethiopia (five study sites encompassing 12 facilities), Kenya (two sites encompassing 12 facilities in Murang’a and 12 facilities in Machakos counties), and Nigeria (two sites encompassing 12 facilities in Kano and 12 facilities in Nasarawa states). Baseline and endline assessments at each site will include 744 women who have recently given birth in the quantitative component. We will interview a maximum of 16 birth companions, 48 health care providers, and eight unit managers quarterly for the qualitative component in each country.

**Discussion:**

Ample evidence supports the contribution of birth companions to positive health outcomes for mothers and newborns. However, limited data are available on effective strategies to improve birth companion coverage and inform scale-up efforts. This trial tests a birth companion intervention package in diverse clinical settings and cultures to identify possible barriers and considerations to increasing uptake of birth companions. Findings from this study may provide valuable evidence for scaling up birth companionship in similar settings.

**Trial registration:**

Trial is registered with ClinicalTrials.gov with identifier: NCT05565196, first posted 04/10/ 2022.

## Background

Ethiopia, Kenya, and Nigeria have experienced large increases in facility deliveries in the past decade. In Ethiopia, facility births increased from 11% to 2011 to 48% in 2019, in Kenya from 43% to 2008 to 61% in 2014 and in Nigeria from 23% to 2013 to 40% in 2018 [1–6]. As the volume of facility births increase, improving and ensuring quality maternity care has become a focus area for the Ministries of Health in Ethiopia, Kenya, and Nigeria [7–9].

The presence of a birth companion during a woman’s labor and birth is a simple and low-cost intervention that can improve both maternal and newborn health outcomes. This kind of support has been referred to by several different names: companion of choice at birth; continuous support during childbirth, labor companion, and emotional support during birth [10]. A birth companion can provide emotional support by continuously being present and by giving praise and reassurance; provide information on how the labor is progressing; provide coping techniques such as breathing appropriately; give comfort such as massage; assist in mobility; give reminders to drink fluids and visit the restroom; advocate on behalf of the woman; and engage in caring for the newborn [11–13].

A 2013 Cochrane systematic review of 21 trials showed that women who had continuous presence and support during labor or labor and birth were more likely to have a spontaneous vaginal birth, and report satisfaction with the care they received. Having continuous support during labor was also associated with shorter labor, reduced need for pain medication during labor, and lower likelihood of having a caesarean delivery or instrumental vaginal birth. Continuous support was also associated with lower rates of depression and improved Apgar scores of the newborn [11].

The evidence that birth companionship improves labor outcomes and experience of care has been available for many years. As such, global guidance supports birth companions. The World Health Organization (WHO) states “continuous companionship during labor and childbirth is recommended for improving women’s satisfaction with services;” [14] and a companion of choice is recommended for all women throughout labor and childbirth [15]. The WHO quality of care framework also states that a woman should have access to the social and emotional support of her choice [16]. National polices in Ethiopia, Kenya, and Nigeria support birth companions. However, birth companions are not yet incorporated into routine practice, and women typically labor and give birth alone [11, 12, 17–23]. One study in Ethiopia showed that 98% of women were not allowed to have a birth companion, and another study in Kenya found 78% were not allowed a companion during labor and 84% during delivery [17, 19]. In Nigeria, a study showed that only 29% of mothers experienced companionship during delivery [20].

A 2019 Cochrane review outlined common barriers to facilities adopting birth companionship: health facility policies, provider attitudes, facility infrastructure, and preparation of families and birth companions [24]. Implementation research on birth companions is needed to test interventions in varying clinical settings and cultures that will address these barriers and lead to increased uptake of birth companions in health centers and hospitals. Common barriers identified in the literature that hinder birth companion presence include lack of facility polices, low knowledge of the benefits of birth companionship by providers and families, space and privacy constraints, negative attitudes of providers, and lack of preparedness of families and birth companions [24]. It is unknown whether a health facility’s readiness to change impacts their ability to successfully implement interventions that increase uptake of birth companions [10].

### Birth companion intervention package

**Fig. 1:**
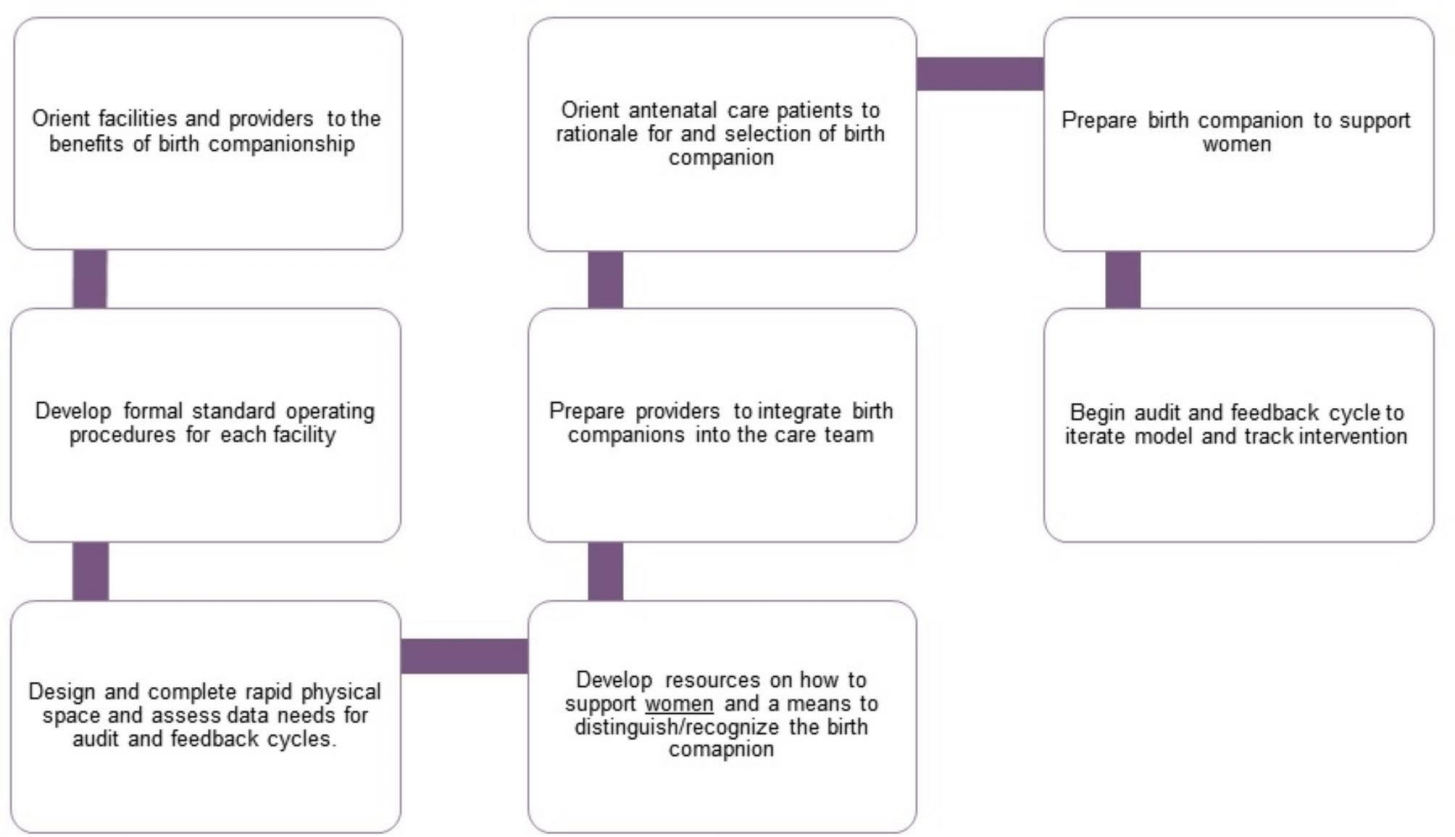
Components of the birth companion intervention package of interventions

This study will evaluate an intervention package of eight elements to increase birth companion coverage based on the framework described in the Cochrane review (Fig. 1) [24]. It will include the following:

1. *Orient facilities and providers to benefits of birth companionship.* Key individuals (e.g., facility managers, clinical staff) from each intervention facility will be oriented on the benefits of birth companions. We will arrange for a benchmark visit to a facility where birth companions have been incorporated into practice, where possible.
2. *Develop/update formal standard operating procedures (SOPs) for implementing birth companions and plan to implement guideline/SOP*. We will develop a template to be provided to each intervention facility along with an existing SOP that can serve as an example. We will identify individuals in these facilities to participate in the development of a SOP and one person to lead the process. A workshop will be conducted to develop the SOP. Participants will include staff, postpartum women with experience of having a birth companions, and birth companions. The SOP will be finalized and endorsed by the facility director or designee.
3. *Design and complete rapid physical space and assess data needs for audit and feedback cycles*. We will develop a template, which, together with the intervention facility staff, will be used to assess and determine the physical/environmental changes or enhancements that are needed to incorporate or increase use of birth companions (e.g., chairs for companions to sit, more functional privacy barriers). We will then support the facilities to make the necessary changes and procure the required equipment and supplies. We will initially assist facilities to assess their data availability to measure coverage of birth companions as part of facilitating audit and feedback cycles. If additional data is required, we will work with the facility to develop methods to measure coverage and then establish targets.
4. *Develop resources on how to support women and a means to distinguish/recognize the birth companion*. We will assess the available birth companion-related resources and either adapt or develop new materials (e.g., posters, brochures, short video). Job aids for care providers will allow them to communicate the benefits of birth companions to pregnant women and clarify the role of providers and birth companions. For mothers, the resources will promote the presence and importance of birth companions. For birth companions, the materials will explain their role during labor, birth, and the postnatal period. We will also assess the best way to designate the birth companions (e.g., gowns, nametags, stickers) and provide the facilities with the necessary materials.
5. *Prepare providers to integrate birth companions into the care team.* An orientation and planning meeting will be conducted to operationalize the SOP with intervention facility staff working in antenatal care (ANC) clinic and labor and delivery ward (including support staff). Community health workers will also be included so they can raise awareness about the birth companion intervention.
6. *Orient ANC clients to rationale for and selection of a birth companion*. After the launch of the intervention, pregnant women anticipating delivery in intervention facilities will be oriented to birth companions during ANC, optimally during their third trimester ANC visit. Similar information will also be available in the labor ward, ensuring that the information reaches women who did not have a third trimester ANC visit at intervention facilities. Pregnant women who are close to their due date will also be reminded by community health workers in the intervention health facilities’ catchment area to plan on having a birth companion. We will use developed materials to communicate the benefits of birth companion.
7. *Prepare birth companions to support women*. Birth companions in intervention facilities will be oriented using the resources developed (e.g., short video) on their role and how they can best support the woman during labor, birth, and the postnatal period. We will also ensure that birth companions are easily recognizable (e.g., gown, wristband, nametag).
8. *Begin audit and feedback cycle to iterate model and track intervention*. We will support existing facility meetings to review data. Facilities will modify implementation of the package, as needed, to increase coverage. We will capture modifications to the intervention through process documentation.

Use of a birth companion will be voluntary for participants and non-participants who seek care at study site facilities. There are no retention strategies for women or companions for the intervention. Staff at intervention facilities will be supported to implement the package through on-site monitoring and audit and feedback cycle data reviews.

## Methods

### Aims

To determine the feasibility and acceptability of the birth companion intervention package to increase coverage of birth companions during labor, birth, and the postnatal periods.

### Design and setting

This study will use a two parallel arm cluster randomized controlled trial (cRCT) design stratified by country/county and birth volume, with 12 public health facilities in Ethiopia, 24 public health facilities in Kenya, and 24 public health facilities in Nigeria. This study will be happening in and near Addis Ababa city of Ethiopia, Machakos and Murang’a counties of Kenya, and Kano and Nasarawa states of Nigeria (Tables 1, 2 and 3).

**Table 1:** Health facilities in Ethiopia by type of facility and the average number of births per month

Facility #	Average # births/ month	Type of facility ^a, b^
1	327	CEmONC
2	262	CEmONC
3	122	BEmONC
4	91	BEmONC
5	83	CEmONC
6	74	BEmONC
7	46	BEmONC
8	29	BEmONC
9	27	BEmONC
10	20	BEmONC
11	16	BEmONC
12	16	BEmONC

**Table 2:** Health facilities in Kenya by county, type of facility, and the average number of births per month Murang’a County

Facility #	Average # births/month	Type of facility ^a, b^
**Machakos**		
1	293	CEmONC
2	194	CEmONC
3	96	CEmONC
4	63	CEmONC
5	48	BEmONC
6	41	CEmONC
7	40	CEmONC
8	39	BEmONC
9	28	BEmONC
10	26	BEmONC
11	18	BEmONC
12	18	BEmONC
**Murang’a**		
1	318	CEmONC
2	287	CEmONC
3	121	CEmONC
4	115	CEmONC
5	106	CEmONC
6	66	CEmONC
7	53	BEmONC
8	32	CEmONC
9	31	BEmONC
10	30	BEmONC
11	28	BEmONC
12	19	BEmONC

^a^ CEmONC: Comprehensive emergency obstetric and newborn care

^b^ BEmONC: Basic emergency obstetric and newborn care

**Table 3:** Health facilities in Nigeria by state, type of facility, and the average number of births per month State

Facility #	Average # births/month		Type of facility ^a,b^
**Kano**			
1	260		CEmONC
2	240		CEmONC
3	179		BEmONC
4	177		CEmONC
5	144		BEmONC
6	143		BEmONC
7	48		BEmONC
8	45		BEmONC
9	39		CEmONC
10	38		BEmONC
11	37		BEmONC
12	34		BEmONC
**Nasarawa**			
1	312	CEmONC	
2	300	CEmONC	
3	135	BEmONC	
4	109	CEmONC	
5	69	CEmONC	
6	64	BEmONC	
7	45	CEmONC	
8	41	CEmONC	
9	37	BEmONC	
10	37	BEmONC	
11	37	CEmONC	
12	36	BEmONC	

We will use a mixed methods approach to assess impact of the birth companion intervention package and measure the study outcomes with exit interviews, health facility register data extraction, in-depth interviews (IDIs), focus group discussions (FGDs), and key informant interviews (KIIs).

There are five data collection periods. We will first conduct a baseline assessment over the course of approximately four weeks, in facilities meeting the inclusion criteria. Health facilities that decline to participate in the study will be replaced by another facility. Facilities will be randomized after the baseline survey in a 3:1 ratio (i.e., for every three facilities that implement the birth companion intervention, one will serve as a control facility). Randomization is stratified by the number of facility deliveries and by country (Ethiopia) or by county or state within a country (Kenya and Nigeria). Intervention facilities will prepare to introduce the birth companion intervention package over the course of approximately two months to facilitate the presence of birth companions during labor, birth, and the postnatal period. Control facilities will continue to provide the local standard of routine care which may or may not include a birth companion. Facilities will not be replaced or reallocated to study arm post-randomization. This study does not place restrictions on facilities or participants regarding participation in other interventions or research.

We will have three quarterly quantitative and qualitative data collection periods, each lasting for approximately one week. We will conduct an endline quantitative survey, approximately 12 months after birth companion intervention initiation. Mothers, birth companions, health care providers, and unit managers in study facilities will have the option to decline to participate in the study.

**Fig. 2:**
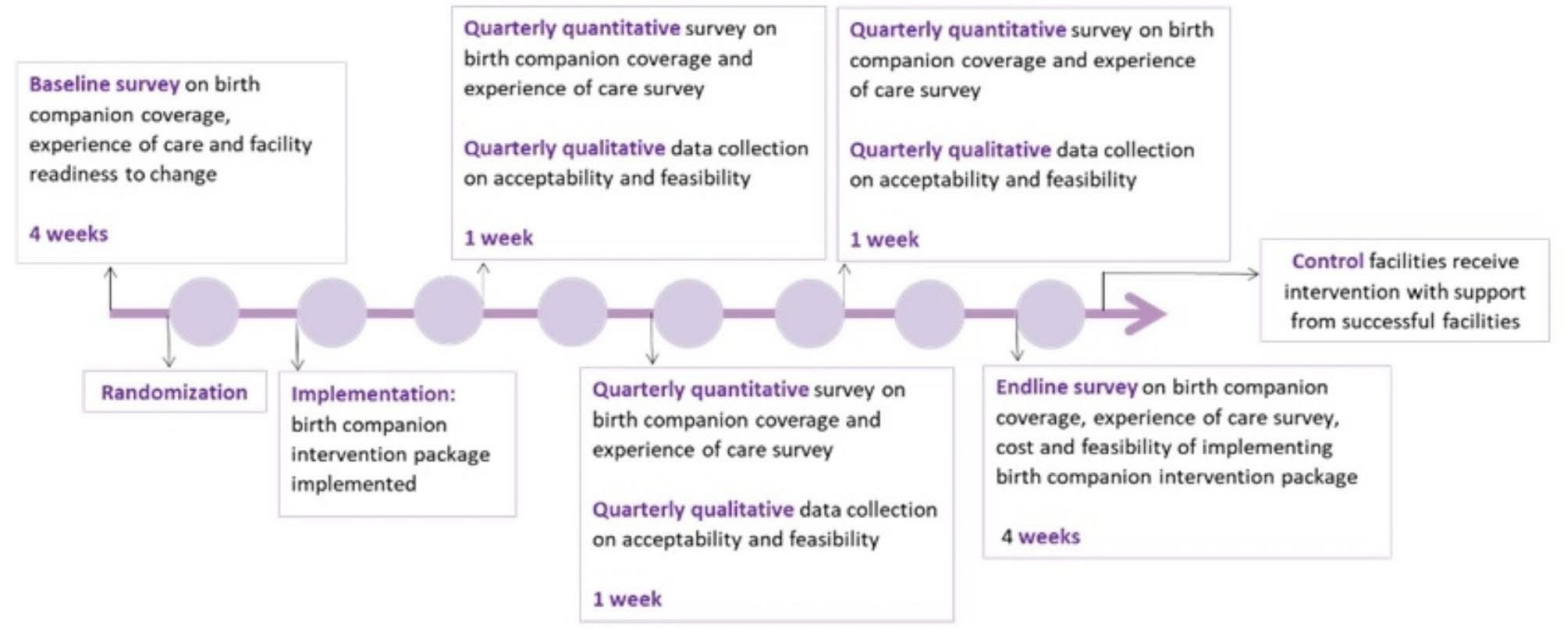
Overview of the study timeline for the birth companion intervention

At the end of the study, control facilities will be supported to implement the birth companion intervention package by staff from an intervention facility that has a birth companion coverage of approximately 50% or higher (see Fig. 2), assuming that one is available. We will provide limited technical and material support during this period.

### Study population

The study population will include: eligible women who give birth at participating facilities; birth companions of eligible women; all eligible providers working in the ANC, and labor, birth, and postpartum wards; and unit managers who work in the facilities during the study period. Inclusion and exclusion criteria are shown in Table 4.

**Table 4:** Inclusion and exclusion criteria for facilities and study participants

Participant	Inclusion	Exclusion
Health facilities	• Prior to randomization, head of facility grants permission for facility to participate in the study • Be willing to develop, adopt, and implement the birth companion standard operating procedures and other components of the birth companion intervention package • Have 16 to 30 births minimum per month during the past three months • Be in the Addis Ababa and surrounding area of Ethiopia; Machakos or Murang’a County of Kenya; or Kano or Nasarawa States of Nigeria	• Facility staff strike or other disturbance to routine care noted prior to randomization that would pose significant challenge(s) to achieving the study objectives
Providers	• At the time of enrollment, work in antenatal care and/or labor and birth ward(s) of study facility • Able and willing to provide informed consent to participate in the study	
Mothers	• Per participant report, age 15 years or older • Have a vaginal birth • Able and willing to provide informed consent to participate in the study	• Unable to participate in an interview due to their physical or emotional condition (e.g., caused by an adverse birth outcome) • Unable to provide valid information because of mental or other serious health condition
Birth companions	• For emancipated minors, age 15 years or older. If not an emancipated minor, 18 years or older. Age per participant report • Person was present at labor and/or birth • Identified as a birth companion by the birthing mother • Willing and able to provide informed consent to participate in the study	• Unable to provide valid information because of mental or other serious health condition
Unit managers	• Involved in the implementation and management of the birth companion intervention • Able and willing to provide informed consent to participate in the study	

### Sample size

#### Quantitative

The sample size justification is based on estimating coverage of birth companionship. We assumed a within-facility intra-class correlation coefficient (ICC) between 0.05 and 0.5 (higher is more reasonable). We also assumed post-birth companion support coverage at 50%. Baseline/standard practice birth companion coverage varied between 5% and 20%.

Sample size is calculated separately by country for two-sample comparison of proportions (with pooled Z-test) in a cluster randomized design using PASS (PASS 15 Power Analysis and Sample Size Software (2017). NCSS, LLC. Kaysville, Utah, USA, ncss.com/software/pass) [25].

The calculations below apply to the study area in Ethiopia, the two study counties in Kenya, and the two study states in Nigeria. We assume the same baseline level of birth companion coverage in all three countries. There are the same total number of facilities to be randomized at each study area, n = 12 (three in the standard arm and nine in the intervention arm). There will be a total of 12 facilities in Ethiopia, 24 in Kenya, and 24 in Nigeria.

The number of women per facility and total number of women across the two study arms per study site needed was calculated for at least 80% statistical power to test differences in proportions of birth companion coverage between study arms at 0.05 level of statistical significance, assuming that for every three facilities randomized to the birth companion intervention, one facility will be randomized to the standard practice arm and a coefficient of variation of one. Post-birth companion intervention coverage is assumed to be 50%.

Conservatively assuming 20% birth companion coverage in the standard practice (control) arm and 50% in the birth companion intervention arm at the endline, to have 80% statistical power to detect this difference in proportions at 0.05 level of statistical significance, coefficient of variation of one, we will need to include 62 women who recently gave birth (on average) at each of the 12 health facilities per study area, for a total of approximately 744 women across the two study arms (186 in control arm, 558 in the intervention arm). In this scenario in each of the study sites (Addis Ababa and surrounding area, Machakos and Murang’a counties, and Nasarawa and Kano states), three facilities will be randomized to the standard arm, and nine facilities to the birth companion intervention arm. Since the coefficient of variation is almost one, based on the facility data presented in Tables 1, 2 and 3, the same sample size is used for considering a varying cluster size. We will include all births occurring within the seven data collection days from all facilities. Thus, we will recruit more births from health facilities with a higher volume of births and fewer from health facilities with smaller volume of births. Randomization of facilities was conducted by the Johns Hopkins Biostatistics Center.

#### Qualitative

For mothers in intervention facilities, we will include both women who reported having and not having a positive experience with their labor and birth care from the quantitative survey. We will assess the acceptability of birth companions by interviewing mothers who had a birth companion in intervention facilities. We will also interview their birth companions, including husbands/partner, other family members, and friends. For providers and unit managers, we will select some staff from intervention facilities where birth companion coverage is high and some from where birth companion coverage was low. We will plan to have up to approximately 16 interviews with mothers and birth companions. We will also have approximately eight FGDs with providers and eight KIIs with unit managers (Table 5).

**Table 5:** Summary of sample size table for qualitative data collection

Respondent type	Methodology	Estimated number of quarterly of data collection in each country
**Mothers who had a birth companion** in intervention facilities	IDIs to understand experience of care, and experience and acceptability of birth companions	Up to approximately 16 interviews
**Birth companions** in intervention facilities	IDIs to understand acceptability of birth companions	Up to approximately 16 interviews
**Providers** in intervention facilities	FGDs to understand acceptability of birth companions	Up to approximately eight discussions
**Health facility unit managers** in intervention facilities	KIIs to understand feasibility and acceptability of birth companions	Up to approximately eight interviews

### Outcomes

#### Primary outcome

1. Proportion of women at study facilities that have a birth companion during labor, birth, and the postnatal period.

#### Secondary outcomes

***Experience of care***

1. Among those who report having a birth companion, proportion who report that the birth companion was the one of their choice.
2. Mothers’ perception of the birth companion experience.
3. Mothers’ score on Person-Centered Maternity Care scale [26], comparing those that have and do not have a birth companion.

#### Feasibility

4. Proportion of providers and unit managers who report the physical environment of care supports the presence of birth companion during labor, birth, and the postnatal period.

#### Acceptability

5. Mothers’, birth companions’, providers’, and unit managers’ perception of birth companions.

#### Exploratory outcomes

6. Score on facility readiness to change.
7. Facility-level costs for updates to physical environment of care and other implementation costs.

### Recruitment and consent process

#### Recruitment

Data will be collected at baseline and approximately quarterly for approximately one year after the birth companion intervention package implementation is introduced in all study facilities. Qualitative data will be collected approximately quarterly at intervention facilities. The recruitment process by study participant type is detailed in Table 6. The recruitment will be done by study staff who will have secondary and post-secondary training.

**Table 6:** Recruitment strategy for study participants

Participant type	Recruitment process
**Mothers**	Quantitative data will be collected from all consenting mothers who give birth in selected/participating facilities during the five data collection periods. Pregnant women coming to the facilities for their ANC visit will be provided with an information sheet so they can learn about the study. Providers will also refer women coming to give birth to the study staff so they can find out more about the study. Investigators will provide information, assess the eligibility of the women, and obtain consent before they give birth. If this is not possible, eligibility and consent will be obtained after birth. A subset of mothers giving birth in intervention facilities will also be asked to participate in the quarterly qualitative assessment prior to discharge. We will use data obtained from that quantitative survey in intervention facilities to select women who did and did not have birth companions, who had different types of birth companions (male/female, partner/non-partner, family member/non-family member) as well as those that were and were not satisfied with the experience of care.
**Birth companions**	Mothers recruited for the quarterly qualitative assessment will be asked if they had a birth companion. If present, investigators will approach the birth companions for recruitment into the study.
**Providers**	Study investigators will work with the facility directors or their designees in intervention facilities to identify providers working in the ANC, labor, and birth wards. Once identified, they will be approached and screened for the quarterly FGDs.
**Unit managers**	Study investigators will work with the facility directors or their designees in intervention facilities to identify unit managers from the ANC, labor, and birth wards. Investigators will approach unit managers and screen them for the quarterly KIIs.

Privacy will be ensured by offering study participation (recruitment) in a space in the facility or outside the facility that allows for audio and visual privacy. We do not expect participation in this study to be associated with any mentioned risks.

#### Consent process

Study staff will have a minimum of secondary and post-secondary training. They will be trained on basic research ethics and the study procedures. The Johns Hopkins School of Public Health (JHSPH) Ethics Field Training Guide will be used during the training.

For all study participants, the information that will be collected by this study team will be kept confidential. The information will not be accessed by anyone except the study team, and it will only be used for this research purpose. No individual identities will be used in any reports or publications resulting from the study. Procedures for consenting study participants are described below.

#### Consenting mothers and birth companions

A data collector will be placed at each facility during the data collection periods. Consent documents will be translated into the local languages. If possible, the data collectors will approach women when they arrive at the facility and screen them to assess their eligibility (if this is not possible, they will be assessed for eligibility after the birth). Informed consent will be obtained after the birth in a private space within the facility. For the qualitative study, women who consent to participate in the study will be asked if they had a birth companion. If present, a similar procedure will be followed for consenting birth companions. In the consent process, potential participants will be informed of why they were chosen, their rights as participants, risks, and benefits. Anyone declining consent will not be included in the study. Surveys and interviews will be administered after consenting the clients. Confidentiality will be kept to the maximum standard during data collection and data handling.

#### Consenting providers and unit managers

Providers and unit managers in ANC, labor and birth wards of intervention facilities will be approached for consent for the duration of the study (approximately 16 months) by study staff. Providers and unit managers will be oriented on the study, informed of why they were chosen, their rights as participants, risks, and benefits. Anyone declining consent will not be included in the study. New providers and unit managers will be consented at the time of data collection. All surveys, interviews and IDIs will be administered after consenting the clients. During the baseline survey, providers will be asked to respond to the facility readiness to change survey. For IDIs and FGDs, they will be approached quarterly after the launch of the intervention. For the quantitative feasibility study, providers in the intervention facilities will be approached during the endline survey. Every effort will be made to protect the confidentiality of participants, but due to practical constraints (e.g., size of facility), it may not be possible to guarantee that supervisors will remain unaware of participation.

### Data collection

#### Quantitative

Pre- and quarterly post-intervention data will be collected from facilities. All women who deliver in the intervention and control facilities during the data collection period and meet the eligibility criteria will be interviewed prior to being discharged. Basic demographic data (age, parity, education, marital status, and socio-economic status) will be collected. Structured questionnaires will be used to assess coverage of birth companion, choice of birth companion (if present) and experience of care. The person-centered maternity score has been validated and is a 30-item scale [26]. It has three sub-scales: (1) dignity and respect, (2) communication and autonomy; and (3) supportive care. Furthermore, we will collect data from providers in all facilitiesto assess the facility readiness to change using a standardized tool. This tool has five context assessment domains: (1) commitment and motivation; (2) ability to implement; (3) internal culture; (4) clinical team functionality; and (5) knowledge and ability to do the intervention. This tool is formulated for health facility staff and leaders. At endline, we will evaluate the feasibility of implementing a birth companion intervention package in intervention facilities. We will also conduct a quantitative analysis at endline to assess the cost associated with implementing the birth companion package.

#### Qualitative

To explore the experience of having a birth companion, we will conduct quarterly interviews with mothers in intervention facilities who meet the inclusion criteria. Acceptability of the birth companion intervention will also be assessed quarterly by conducting IDIs with both women who had a birth companion and their birth companions in intervention facilities. Specific topics to be explored with mothers include: their awareness of the birth companion concept; support/advice they received about having a birth companion; their choice and reason for choosing the birth companion; the type of support their birth companion provided; and their experience of/satisfaction with the care from providers in the presence of birth companion. The following domains will be explored with birth companions including their experience of being a birth companion; how they provided support to mother; their self-reported preparedness to be a birth companion; their perceptions of the orientation materials (e.g., videos, posters, and brochures); and the means used to designate them as a birth companion.

To assess the acceptability as well as feasibility of the birth companion intervention from the supply side, we will conduct FGDs with providers in labor and birth wards and KIIs with unit managers who meet the eligibility criteria. FGDs with providers at intervention facilities will include: their experience of being oriented on the birth companion concept; the materials provided for supporting the birth companion intervention; the physical and managerial level support of the birth companion intervention; and, their experience of and attitudes towards having a birth companion during labor, birth and the postnatal period; perceived changes in their communication and relationship with clients (if any); changes in their workloads; sustainability of having birth companions; and, suggested changes to the model required to offer birth companions. KIIs will be conducted with unit managers in intervention facilities to explore the processes, and where present, challenges of developing birth companion SOP; care provider’s orientation; facility adjustments to facilitate birth companions; integrating birth companions into the care provided; and monitoring the birth companion coverage.

### Statistical analysis and power

The following methods will be used to describe group characteristics or differences: for categorical variables, the number, and percent in each category; for continuous variables, the mean, median, standard deviation, quartiles, and range (minimum, maximum). Adequacy of the randomization will be assessed at baseline by comparing characteristics of participants and facilities in intervention and control arms, including demographics, birth companion coverage, and experience of care.

### Analysis of primary outcomes

Proportion of mothers whose births included birth companions will be reported and compared between intervention and standard care arms at the end of the intervention using cluster-weighted chi-square test [25, 27]. If the baseline data shows difference between intervention and standard care arms in covariates which may affect the outcome, such covariates will be adjusted for using logistic regression model with cluster-correlated standard error estimates to account for the correlation of outcomes within facility.

The model will include birth companion status at birth as the study outcome, study arm as the primary predictor, and adjustment for covariates that are differentially distributed between the study arms. The primary analysis will be conducted separately for each sub-national region.

### Analysis of secondary outcomes

#### Quantitative

For the binary secondary quantitative outcomes, we will use similar analytical methods as for the primary outcome. For continuous outcomes, such as facility readiness for change or score on the Person-Centered Maternity Care scale, we will use a cluster-weighted t-test. As in the case of the primary outcome, linear regression models with cluster-correlated standard error estimates will be used to compare the study arms while adjusting for covariates that are differentially distributed between the study arms.

If the proportion of missing data on these covariates is more than 5%, we will use statistical imputation for missing data. Similarly, if the proportion of missing outcome data is small (< 5%), then complete-case analyses will be used. If the amount of missing data for the study outcomes is > 5% or if it is suspected that missingness for the outcome data are not completely at random, based on examination of reasons for missingness, a series of sensitivity analyses for the distribution of missing outcome data will be performed.

#### Qualitative

Audio recordings of interviews and FGDs will be transcribed from the local language to English by individuals proficient in the local language and English. The transcriptions will be spot checked for quality against the recordings by study staff conversant in both English and local languages used. Thematic analysis will be employed. As qualitative research is a learning experience in the field, emerging themes in the field shall be considered for inclusion in subsequent data collection activities after discussion by senior research management at Addis Continental Institute of Public Health (ACIPH), International Center for Reproductive Health Kenya (ICRHK), and Maternal and Reproductive Health Research Collective (MRHRC). Analysis will be on-going. Coding of textual passages will be done in Atlas-ti or other qualitative data analysis software. Qualitative data analysts will have training and experience in coding of textual information on health topics. The emerging themes will be summarized in tables and other appropriate formats.

### Data management

#### Quantitative data

Data collectors will receive in-depth training on the study objectives, procedures, and ethics by ACIPH, ICRHK, and MRHRC. A detailed manual with SOPs will be prepared and used in training, piloting, and research. The training will also include proper use of tablets and SurveyCTO, which will be used for data entry at the point of collection. There will be daily checks for completeness and consistency of the collected data. The electronic data capturing system will limit data inaccuracies by building in logic, range, and skip-patterns, and minimizing outliers. Investigators will also be supported with ongoing supervision visits from ACIPH, ICRHK and MRHRC.

Data will be de-identified after data collection by ACIPH, ICRHK, and MRHRC and shared with John Hopkins Biostatistics Center for multi-country analysis. De-identified data will be archived according to Bill & Melinda Gates Foundation policies on Synapse or a similar platform. We will also adhere to ACIPH, ICRHK and MRHRC data sharing policies.

#### Qualitative data

ACIPH, ICRHK and MRHRC will also provide training on study objectives, step-by-step training procedures and guidelines of data collection, recording, and transcribing recordings, and ethics of data collection. Audio recordings of interviews and FGDs will be randomly selected and compared against transcripts for data quality monitoring.

No data or safety monitoring oversight is planned for this study. External audits will occur if requested by local regulatory entities. Data security will be carefully and continuously maintained, with limited secure access to data. All study data collection and administrative forms will be identified by coded number to maintain participant confidentiality. All records that contain names or other personal identifiers, such as locator forms and consent forms, will be stored securely. All local databases will be secured with password protected access systems. To the extent possible, we will minimize risks to confidentiality during data collection by directly entering data into the secure database without the use of paper forms. Participant confidentiality will be ensured during FGDs and KIIs by conducting interviews in a secure location, preferably a closed-door room in a health facility, or, in the case of KIIs, within the participants’ offices.

Quarterly study findings will be shared with health care providers. The final study results will be published as scientific articles and reports. No professional writers will be used. Publications arising from this evaluation will follow the recommendations from the International Committee of Medical Journal Editors.

#### Harms

We do not anticipate that this study will result in adverse events. If any unanticipated adverse event or problems occur, the PI will report such occurrences to the JHSPH Institutional Review Board (IRB) and to the local IRBs in Ethiopia, Kenya, and Nigeria, with all reporting and response according to IRB policies and recommendations.

## Discussion

This protocol describes the birth companion intervention and the research in Ethiopia, Kenya, and Nigeria. Despite strong evidence and global recommendation in support of birth companions, many women continue to give birth alone in unfamiliar, and often poor quality, facility settings. This study of a low-cost intervention package will provide important evidence for overcoming common barriers within hospitals and health centers that currently discourage or prevent women from having a birth companion.

### Trial status

This trial is registered with ClinicalTrials.gov with identifier: NCT05565196, first posted October 4, 2022. This trial is currently recruiting. Baseline surveys were conducted in Ethiopia October 10─November 22, 2022 and in Kenya from November 1─December 9, 2022. For Nigeria, we have received ethical clearance and are preparing to conduct the baseline survey. We anticipate reaching study completion in mid- to late-2024.

### Protocol

Version 1.6, September 2023.

## Data Availability

No data is associated with this article. Study data collection tools and protocol can be made available upon reasonable request. Data availability is anticipated to begin on August 1, 2025. All requests should be sent to arc@jhpiego.org.

## Abbreviations

ACIPH: Addis Continental Institute of Public Health
ANC: Antenatal care
Crct: Cluster randomized control trial
FGD: Focus group discussion
ICC: Intra-class correlation coefficient
ICRHK: International Center for Reproductive Health Kenya
IDI: In-depth interview
IRB: Institutional review board
JHSPH: John Hopkins School of Public Health
KII Key: informant interview
MRHRC: Maternal and Reproductive Health Research Collective
PI: Principal investigator
SOP: Standard operating procedure
WHO: World Health Organization
